# “Supporting” Players Take the Lead in Protecting the Overstimulated Brain

**DOI:** 10.1371/journal.pbio.0040371

**Published:** 2006-10-17

**Authors:** Liza Gross

For many years, astrocytes got no respect. These star-shaped cells were long considered mere space fillers, providing structural support to buttress their betters. It’s now clear that astrocytes play an active role in brain function. With their octopus-like protrusions, called processes, astrocytes remove neurotransmitters from neuron synapses, regulate the chemical composition of the extracellular environment, and can influence neuronal activity. And now a new study by Christel Genoud, Graham Knott, and colleagues provides further evidence that astrocytes take a proactive role in brain function, by showing that alterations in cortical activity cause changes in the physical interactions between astrocytes and neurons. These changes could facilitate the uptake of potentially damaging excess neurotransmitters.

Brain signals travel down the axon of a transmitting, or “presynaptic,” neuron as an electrical impulse. The electrical signal is converted into a chemical signal (neurotransmitter) when the impulse reaches the presynaptic nerve terminal (or bouton). Neurotransmitters carry the signal across the gap between neurons, called the synaptic cleft, to a dendrite of the receiving, “postsynaptic” neuron. Interactions between the bouton and specialized postsynaptic protrusions in dendrites, called dendritic spines, mediate synaptic transmission.

Clearing glutamate from the synaptic cleft may be astrocytes’ most critical function. The principal neurotransmitter in the brain, glutamate plays a major role in sensory perception. But too much glutamate causes trouble: as an excitatory neurotransmitter, it can stimulate the postsynaptic neuron until it is reabsorbed by membrane-bound transporters in the transmitting neuron or by transporters in astrocytes. Overstimulation can damage the nervous system, causing seizures and even stroke.

Astrocytes take up glutamate with two transporters—glutamate transporter 1 (GLT1) and glutamate aspartate transporter (GLAST)—and corral excess glutamate with their abundant processes. (Neuronal transporters recycle neurotransmitters for later use.) Genoud et al. wondered whether increased neuronal activity in the sensory cortex would trigger a corresponding response in astrocytes.

To find out, the authors hyperstimulated a single whisker in unanesthetized adult mice, a protocol that triggers physiological and morphological changes in the region of the sensory cortex that receives information from the stimulated whisker. Adult mice received 24 hours of continuous whisker stimulation The authors then located and removed the corresponding region of the cortex. Tissue was extracted immediately after stimulation from one group and four days later from another stimulated group.

After 24 hours of whisker stimulation, levels of both astrocyte glutamate transporters, GLT1 and GLAST, more than doubled. Four days after stimulation had stopped, transporter levels returned to those seen in the unstimulated controls. The researchers also analyzed levels of the neuronal glutamate transporter EAAC1 in a separate group of animals but found that stimulation had little effect on its expression.

**Figure:**
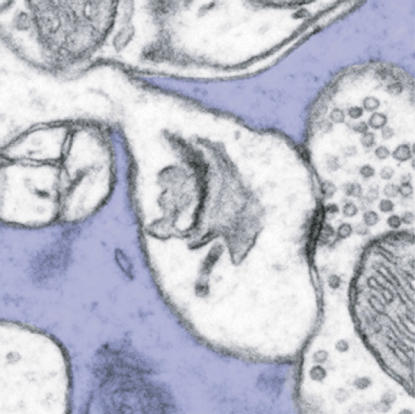
Electron micrograph of a dendritic spine synapsing with an axonal bouton and ? anked on either side by an astrocytic process (blue).

Using electron microscopy and serial sections of the stimulated brain samples, the authors used high-resolution images to generate 3D reconstructions of the contacts between astrocytes and adjacent bouton-dendritic spine pairs. These reconstructions showed that stimulation caused a marked increase in the percentage of spines with their excitatory synapses “completely occupied” by astrocytes. Although stimulation does not increase contact between dendritic spine and bouton, it does rouse astrocytes to encircle the interacting synapses, where glutamate is transmitted.

Both stimulation-triggered changes in astrocyte shape and behavior—glomming onto excitatory synapses and upregulating their glutamate transporters—help clear glutamate from the synaptic cleft. Through flexible, dynamic interactions with neurons, the authors conclude, astrocytes prevent the accumulation of glutamate, which is most likely to occur during sensory overstimulation. This study demonstrates yet another functional benefit of astrocyte-neuron interactions, but the authors expect to find more. Given astrocytes’ control over synaptic transmission and evidence that they can release their own neurotransmitters, it seems these long-misunderstood cells are finally getting their just desserts.

